# Analysis of 25(OH)D Serum Concentrations of Hospitalized Elderly Patients in the Shanghai Area

**DOI:** 10.1371/journal.pone.0090729

**Published:** 2014-03-05

**Authors:** Xudong Mao, Hongchao Zheng, Zhiwen Liu, Yan Wu, Risu Na, Chunping Wang, Xulei Zheng, Jing Gao, Liming Wu, Xiaohong Shi, Cong Liu, Hongguang Sheng

**Affiliations:** 1 Department of Endocrinology, Xuhui District Central Hospital, Shanghai, China; 2 Department of Cardiology, Xuhui District Central Hospital, Shanghai, China; 3 Department of Psychiatry, Shanghai Mental Health Center, Shanghai, China; 4 Department of Information, Xuhui District Central Hospital, Shanghai, China; University Heart Center Freiburg, Germany

## Abstract

**Objective:**

To find an association between basic characteristics, seasons as well as disease types and 25-Hydroxyvitamin D serum concentrations in Chinese patients.

**Methods:**

We randomly selected 5470 Chinese patients with various diseases, who were hospitalized between May 2012 and August 2013 in Shanghai and analyzed their serum 25-Hydroxyvitamin D2 (25 (OH)D2) and 25-Hydroxyvitamin D3 (25(OH)D3) concentrations with liquid chromatography-tandem mass spectrometry (LC-MS/MS) as well as their parathyroid hormone (PTH) and serum creatinine blood levels. The resulting data were analyzed by linear regression and variance analyses or multivariate analysis with covariance.

**Results:**

The 25(OH)D serum concentrations were lowest in December. Among the subjects with a median age of 83.0±16.0, the median 25(OH)D2, 25(OH)D3 and total 25(OH)D serum concentrations were 1.00±1.80 ng/ml, 12.20±8.50 ng/ml and 14.80±9.80 respectively, indicating a prevalent 25(OH)D deficiency. According to our multivariate analysis of covariance, the factors affecting 25(OH)D2 and 25(OH)D3 serum concentrations included age, creatinine, PTH, season and type of disease, whereas gender correlated only with 25(OH)D2 and 25(OH)D2 and D3 values correlated negatively with each other. Our results further revealed that 25(OH)D3 levels were low while 25(OH)D2 levels were high among patients with lung diseases, dyskinesia and coronary heart diseases. In addition, participants with diabetes and cerebral infarction had higher 25(OH)D3 serum concentrations compared with lung disease patients.

**Conclusion:**

Vitamin D intake particularly during winter and summer seasons is important especially for elderly lung disease, dyskinesia and coronary heart disease patients to improve their quality of life.

## Introduction

Vitamin D is a secosteroid, which is formed in the skin upon UV-light exposure as Vitamin D3 or is ingested by diet as Vitamin D2. After conversion into its active form calcitriol via calcifediol (25 (OH)D) by the liver and kidneys it is a ligand for the vitamin D receptor (VDR), which forms a heterodimer with the retinoid-X receptor in its activated form and acts as a nuclear hormone receptor transcription factor in many tissues throughout the body including cardiomyocytes, [1] vascular smooth muscle and endothelium.[2], [3] Beside its function as primary regulator of calcium and phosphorus homeostasis for bone mineralization by promoting calcium absorption in the intestines [4], Vitamin D is involved in maintaining the innate immunity balance, muscle (both skeletal and smooth) function and endothelial cell proliferation.[5] It has been reported that Vitamin D deficiency is associated not only with skeletal disease, rickets and osteomalacia but also with cancers, cardiovascular diseases, multiple sclerosis, rheumatoid arthritis, and type 1 diabetes mellitus.[6] In addition, vitamin D leads to enhanced cellular defense against tuberculosis infections because of its monocyte and macrophage activation.[7], [8], [9] According to a global study of vitamin D deficiency in 2009, serum 25(OH)D insufficiency with a concentration below 30 ng/mL (75 nmol/L) is prevalent among the populations studied in every region.[10] Another study also found that 25(OH)D insufficiency (serum 25(OH)D level <30 ng/mL) occurs in almost 50% of the populations worldwide and the prevalence of deficiency (serum 25(OH)D level <10 ng/mL) ranges from 5% to 25% in different regions.[11] Current International Osteoporosis Foundation guidelines recommend a concentration of 30 ng/mL 25(OH)D (75 nmol/L), [12] which is associated with maximal suppression of parathyroid hormone (PTH), and defined vitamin D insufficiency as 25(OH)D levels less than 20 ng/mL (50 nmol/L) and deficiency as levels less than 10 ng/mL (25 nmol/L). Shin et al. reported and evaluated an automated immunoassay for measuring total serum 25(OH)D concentrations and assessed the vitamin D status in an adult Korean population. They found that the prevalence of 25(OH)D deficiency, using cut-off values of <50 nmol/l (<20 ng/ml), was 70.3% in males and 86.4% in females respectively. In addition, the prevalence of vitamin D deficiency was higher in the younger than in older population group (P<0.001). Besides, serum 25(OH)D level changes seasonal with peaks in September and lowest values in February.[13] Although, there are various methods to measure serum 25(OH)D, such as radio-immunoassay (RIA) and high-performance liquid chromatography (HPLC), LC-MS/MS is considered the standard method for serum or plasma 25(OH)D analyses.[13] In this study, we performed LC-MS/MS 25(OH)D serum concentration measurements of hospitalized elderly Chinese patients in Shanghai in order to investigate the prevalence of vitamin D deficiencies and its association with other diseases, especially coronary heart disease, diabetes, hypertension as well as a variety of tumors.

## Subjects and Methods

### Subjects

For this single center study, we randomly selected 5470 patients from 12 endemic areas in the medical department and 6 endemic areas belonging to geriatrics with various diseases, who were hospitalized between May 2012 and August 2013. Exclusion criteria were hepatic insufficiency, renal inadequacy (creatinine≤110), malignancies, hyperthyreosis and hypothyroidism, parathyroid hyperfunction and insufficiency as well as steroid treatments. All subjects had not taken any vitamin D supplement or related prescriptions for at least three months prior to their participation. Written informed consent was obtained from all patients and the study was approved by the Human Research Ethics Committees of the Xuhui District Center hospital of Shanghai (07/40).

### Methods

Patients' empty stomach blood serum samples were kept frozen at −80°C until analysis. Plasma PTH was determined by an IMMULITE 1000 Immunoassay System (Siemens Healthcare Diagnostics, Germany) and liver function enzymes as well as creatinine were measured by using clinical chemistry laboratory tests. Serum 25(OH)D3 and 25(OH)D2 concentrations were analyzed with a Shimadzu series HPLC (Shimadzu Corporation, Japan) coupled to a API 4000 LC-MS/MS system (Applied Biosystems Inc., USA). Data were analyzed using Analyst 1.5 analytic software. The accuracy of lower, middle and higher concentrations was confined between 85% and 115%, with precision deviation below 15%.

### Data analysis

All classified variables were counted by frequency. Continuous variables were described by mean ± standard deviation if following normal distribution and otherwise by median ± interquartile range. We used a variance analysis (ANOVA) when comparing continuous and classified variables (e.g. age) and performed multiplicity analyses using covariance analysis test (ANCOVA). All statistical analyses were performed with SPSS 13.0 software.

## Results

The age range of the 5470 patients was from 21 to 101 years (83.0±16.0) and 47.5% of the participants were between 80 and 89 years old while only 9.3% were under 60. Among them, 2710 (49.5%) were male and 2760 (50.5%) female. The median levels of 25(OH)D2 and 25(OH)D3 were 1.00±1.80 ng/ml and 12.20±8.50 ng/ml respectively, with a median concentration of 13.21±7.98 ng/ml total 25(OH)D. The median values of creatinine and PTH were 70.00±33.00 µmol/L and 43.20±30.90 pg/ml respectively. The diseases included in this study are listed in Table 1. Our results indicated that the predominant form of serum 25(OH)D was 25(OH)D3 and the median 25(OH)D blood concentration in hospitalized elder patients was below 15 ng/ml implying a prevalent Vitamin D insufficiency (Table 1). As visible in Table 2, serum creatinine concentrations varied significantly in gender and age groups as well as in the seasons, whereas PTH concentrations only varied in the age groups.

**Table 1:** Basic Demographic data.

		Frequency (%) or Mean±SD
Gender	Male	2710 (49.5)
	Female	2760 (50.5)
Age	Median ± range interquartile	83.0±16.0
Age group	<40	32 (0.6)
	40–49	67 (1.2)
	50–59	408 (7.5)
	60–69	1196 (12.6)
	70–79	2047 (15.6)
	80–89	4644 (47.5)
	≥90	826 (15.1)
Season	Spring	1241 (22.7)
	Summer	1375 (25.1)
	Fall	1628 (29.8)
	Winter	1226 (22.4)
Clinical Diagnosis	Coronary Heart Disease	1231 (22.5)
	Dyscinesia	504 (9.2)
	Diabetes	826 (15.1)
	Hypertension	757 (13.8)
	Cerebral infraction	420 (7.7)
	Lung Diseases	441 (8.1)
	Others	1291 (23.6)
Creatinine	Median ± range interquartile	70.00±33.00
PTH	Median ± range interquartile	43.20±30.90
25(OH)D2	Median ± range interquartile	1.00±1.80
25(OH)D3	Median ± range interquartile	12.20±8.50
25(OH)D2/3	Median ± range interquartile	14.80±9.80

**Table 2:** The distribution of serum creatinine and parathyroid hormone in gender, age and seasons.

		Serum creatinine			Parathyroid hormone		
		Median±Interquartile range	F value	P value	Median±Interquartile range	F value	P value
Gender	Male	81.0±34.0	312.82	<.0001	43.6±31.4	0.69	0.4076
	Female	62.0±23.0			42.8±30.1		
Age	<40	63.5±24.0^a, b, c^	21.86	<.0001	34.7±22.55^a^	7.07	<.0001
	40–49	57.0±22.0^d^			36.4±26.5^a, b^		
	50–59	62.0±22.0^c, d^			37.5±24.1^b^		
	60–69	62.0±25.0^b, c, d^			43.4±26.0^a, b^		
	70–79	69.0±29.0^a, b^			44.4±30.2^a, b^		
	80–89	74.0±36.0^a, b^			43.9±31.5^a, b^		
	≥90	77.0±42.0^a^			43.7±42.8^a, b^		
Season	Spring	73.0±34.0^a^	5.26	0.0013	42.6±32.8	0.59	0.6224
	Summer	71.0±36.0^a, b^			43.9±29.3		
	Fall	69.0±31.0^b^			43.8±29.05		
	Winter	69.5±34.0^a^			42.3±33.2		

§ indicates containing sub-groups, which are marked with a, b and c. Different letter marks indicate significant differences between the subgroups, same letters indicate no significant differences between the same marked subgroups.

### 25(OH)D2 level in different subject groups

Serum 25(OH)D2 concentrations were not different between gender groups and seasons but varied in different age groups. The 25(OH)D2 serum concentrations were highest in the patients above 90 years old (1.40±3.50) followed by the 80–89 years old group (1.2±2.10) and the patients under 40 years old (0.60±1.30). Furthermore, the 25(OH)D2 levels in patients with cerebral infarction was the lowest (0.70±1.00), while in patients with coronary heart and lung diseases they were fairly high (1.30±2.50, 1.20±2.60) (Table 3). A regression analysis revealed, that 25(OH)D2 serum concentrations positively correlated with patients' age (P<0.0001) and creatinine values (P = 0.0068), while they negatively correlated with PTH and 25(OH)D3 levels (P<0.0001) (Table 4). In a multivariate analysis, factors that influenced 25(OH)D2 blood concentrations included creatinine, PTH and 25(OH)D3 levels as well as age, gender, season and kind of disease (Table 5).

**Table 3:** Univariate analyses of correlations between 25(OH)D2 as well as 25(OH)D3 serum concentrations and subgroups.

Subgroups			Median±interquartile range	F Value	*P* Value
Gender	25(OH)D2	Male	1.00±1.80	2.88	0.0896
		Female	1.00±1.80		
	25(OH)D3	Male	12.20±8.25	0.96	0.3276
		Female	12.20±8.90		
Age Group§	25(OH)D2	<40^a^	0.60±1.30	42.52	<0.0001
		40–49^a,b^	0.80±0.80		
		50–59^a,b^	0.80±0.90		
		60–69^a,b^	0.80±1.00		
		70–79^a,b^	0.80±1.20		
		80–89^b^	1.20±2.10		
		≥90^c^	1.40±3.50		
	25(OH)D3	<40^a^	13.30±8.80	40.02	<0.0001
		40–49^a^	12.25±8.90		
		50–59^a^	14.45±8.35		
		60–69^a^	13.90±8.80		
		70–79^a^	13.80±9.30		
		80–89^a^	11.90±7.80		
		≥90^b^	9.70±8.80		
Season	25(OH)D2	Spring	1.10±1.70	2.65	0.0570
		Summer	1.10±1.80		
		Fall	1.00±1.90		
		Winter	0.90±1.70		
	25(OH)D3	Spring^a^	12.10±7.90	13.71	<0.0001
		Summer^a^	11.80±8.30		
		Fall^b^	13.35±9.20		
		Winter^a^	11.80±7.90		
Clinical Diagnosis§	25(OH)D2	Coronary Heart Disease^a^	1.30±2.50	26.91	<0.0001
		Dyscinesia^b^	0.80±0.90		
		Diabetes^b^	0.80±1.10		
		Hypertension^c^	1.10±1.80		
		Cerebral infarction^b^	0.70±1.00		
		Lung Diseases^c^	1.20±2.60		
		Others^a^	1.10±2.00		
	25(OH)D3	Coronary heart disease^a^	11.70±8.10	28.11	<0.0001
		Dyscinesia^a^	11.00±6.20		
		Diabetes^b^	14.50±10.90		
		Hypertension^c^	13.00±8.00		
		Cerebral infarction^a, c^	12.00±8.30		
		Lung disease^d^	10.10±7.80		
		Others^c^	11.90±8.80		
Coronary Heart Disease	25(OH)D2	Yes	1.30±2.50	33.05	<0.0001
		No	0.90±1.50		
	25(OH)D3	Yes	11.70±8.10	24.88	<0.0001
		No	12.35±8.70		

§ indicates containing sub-groups, which are marked with a, b and c. Different letter marks indicate significant differences between the subgroups, same letters indicate no significant differences between the same marked subgroups.

**Table 4:** Regression analysis correlations between 25(OH)D2 and 25(OH)D3 serum concentrations and age, creatinine, PTH and 25(OH)D2/25(OH)D3 values.

Factors		Regression coefficient	Regression coefficient SE	*P* Value
Age (years)	25(OH)D2	0.07172	0.00528	<0.0001
	25(OH)D3	−0.10089	0.00799	<0.0001
Creatinine	25(OH)D2	0.00383	0.00141	0.0068
	25(OH)D3	−0.00696	0.00213	0.0011
PTH	25(OH)D2	−0.00750	0.00178	<0.0001
	25(OH)D3	−0.03406	0.00266	<0.0001
25(OH)D3	25(OH)D2	−0.13390	0.00886	<0.0001
	25(OH)D3	−0.30520	0.02020	<0.0001

**Table 5:** Multivariate analysis of factors that affect 25(OH)D2 and 25(OH)D3 serum concentrations.

Factors		F Value	*P* Value
Age	25(OH)D2	68.74	<0.0001
	25(OH)D3	79.54	<0.0001
Creatinine	25(OH)D2	6.02	0.0141
	25(OH)D3	6.56	0.0104
PTH	25(OH)D2	69.01	<0.0001
	25(OH)D3	191.89	<0.0001
25(OH)D3	25(OH)D2	214.18	<0.0001
25(OH)D2	25(OH)D3	214.27	<0.0001
Gender	25(OH)D2	5.10	0.0240
	25(OH)D3	-	>0.05
Season	25(OH)D2	3.69	0.0114
	25(OH)D3	15.53	<0.0001
Clinical diagnosis	25(OH)D2	14.90	<0.0001
	25(OH)D3	24.94	<0.0001

### 25(OH)D3 serum concentrations in different subject groups

Similar to 25(OH)D2, 25(OH)D3 serum concentrations did not vary between genders, but the age played a critical role. In contrast to 25(OH)D2, 25(OH)D3 serum values of patients over 90 years old were the lowest (9.70±8.80) followed by the 80–89 years old group (11.90±7.80) and the 50–59 years old group with the highest concentration (14.45±8.35) and the differences were statistically significant (P<0001). In addition, serum 25(OH)D3 levels were significantly higher in spring and fall than in summer and winter (P<0001). Table 6 shows seasonal correlations of single disease groups and their 25(OH)D3 serum concentrations. Beside diabetics, all other patients with the listed diseases had significant or close to significant lower 25(OH)D3 serum levels in summer and winter compared to spring and fall. The 25(OH)D3 serum concentration was lowest in lung disease patients (10.10±7.80ng/ml), followed by dyskinesia and coronary heart disease patients. The serum 25(OH)D3 level of diabetics was the highest (14.50±10.90ng/ml), followed by hypertension patients (Table 3). Similar to 25(OH)D2, the factors which correlated with 25(OH)D3 levels included creatinine, PTH and 25(OH)D2 levels as well as age, season and type of disease in a multivariate analysis (Table 5) and correlated with age, creatinine, PTH and 25(OH)D2 serum concentrations in a regression analysis (Table 4).

**Table 6:** The difference of 25(OH)D3 serum concentrations in different disease and season groups.

Diseases	Spring and Fall		Summer and Winter		Spring and Fall *vs*. Summer and Winter	
	Mean±SD	median±interquartile range	Mean±SD	Median±Interquartile range	t value	P value
Coronary heart disease	13.06±6.37	12.10±8.00	12.38±6.18	11.30±7.80	1.88	0.0602
Dyskinesia	13.19±6.35	11.70±6.60	11.9±5.51	10.60±5.00	2.42	0.0158
Diabetes	15.93±8.3	14.40±10.40	16.17±8.59	14.60±11.20	0.39	0.6947
Hypertension	14.83±6.89	13.60±7.70	13.78±6.7	12.50±7.90	2.10	0.0363
Cerebral infarction	14.24±8.77	12.10±9.60	12.81±5.93	11.80±7.30	1.88	0.0607
Lung diseases	12.26±8.06	10.60±8.70	10.94±6.06	9.75±6.90	1.93	0.0542
Other diseases	14.36±7.21	13.20±8.90	12.64±6.88	11.45±8.10	4.19	<.0001

### Total 25(OH)D serum concentrations in different subject groups

Since 25(OH)D2 level and 25(OH)D3 level are negatively correlated with each other, we next investigated which factors were related to the total 25(OH)D values. According to the results, the total 25(OH)D serum concentration changes were consistent with 25(OH)D3 and negatively correlated with 25(OH)D2. On the other hand, total 25(OH)D levels were highest in patients between 50 and 59 years old, while patients in the 40–49 years and over 90 years old groups had lowest values, but the age related differences were not statistically significant.

From the seasonal aspect, the total 25(OH)D value slightly differed from 25(OH)D3 levels with a peak in fall and low serum concentrations in winter. Consistent with 25(OH)D3 the total 25(OH)D levels were lowest in patients with dyskinesia and lung diseases, but did not significantly correlate with coronary heart disease, which was the most significant discrepancy between total 25(OH)D and 25(OH)D3 levels (Table 7).

**Table 7:** Univariate analysis of correlations between subgroups and their total serum 25(OH)D concentrations.

Factors		Median±interquartile range	F Value	*P* Value
Gender	Male	14.70±9.25	3.78	0.0519
	Female	14.80±10.20		
Age§	<40^a^	15.00±8.30	4.95	<0.0001
	40–49^a^	13.70±8.90		
	50–59^a^	16.05±8.50		
	60–69^a^	15.60±9.10		
	70–79^a^	15.80±10.60		
	80–89^a^	14.50±9.30		
	≥90^a^	13.40±12.60		
Season§	Spring^a^	14.50±9.10	12.56	<0.0001
	Summer^a^	14.40±10.10		
	Fall^b^	16.20±10.10		
	Winter^c^	13.80±9.00		
Clinical Diagnosis§	Coronary heart disease^a^	14.65±10.00	21.85	<0.0001
	Dyscinesia^b^	12.80±6.50		
	Diabetes^c^	16.40±11.00		
	Hypertension^a, c^	15.70±8.80		
	Cerebral infarction^b^	13.60±9.40		
	Lung diseases^b^	12.70±9.50		
	Others^c^	15.60±10.60		
Coronary heart diseases	Yes	14.80±9.60	1.19	0.2757
	No	14.65±10.00		

§ indicates containing sub-groups, which are marked with a, b and c. Different letter marks indicate significant differences between the subgroups, same letters indicate no significant differences between the same marked subgroups.

## Discussion

Vitamin D, obtained either from food or in the skin is firstly transformed by 25-hydroxylase to 25-hydroxyvitamin D. In succession, the intermediate product, 25-hydroxyviatmin D is transported to the kidneys and converted to the biological active form of vitamin D 1,25-dihydroxyvitaminvitamin D under the catalysis of 1α-hydroxylase. Because the half-life time of vitamin D is only 5–7 days, the level of “raw” vitamin D is only accurately reflecting the amount of vitamin D obtained from diet or in the skin. Nevertheless, because the half-life period of 25-hydroxyviatmin D is 20–30 days, it is a proper indicator of patients' vitamin D status. The average 25(OH)D serum concentration in our study was 14.80±9.80 ng/ml, which was less than a previously reported value for healthy adult citizens in Shanghai of 16.88±12.65 ng/ml for participants aged between 50 and 69 and 20.02±16.37 for participants ≥70 years old [14]. Although some institutions recommend cut-off values for deficiency and insufficiency, the clinical correlations for these cut-off values are still not clear.[15], [16] According to the US Endocrine Society guideline, vitamin D deficiency is defined as serum levels less than 50 nmol/l (<20 ng/ml) and insufficiency as serum levels less than 72.5 nmol/l (≤29 ng/ml) [15]. We suspect that the cut-off value employed by the US Endocrine Society guideline for Caucasian patients may not be clinical appropriate for Asian patients. Thus in the present study, we considered 25(OH)D serum concentrations less than 15 ng/ml as deficiency, while levels <10–15 ng/mL is commonly adopted as the definition of vitamin D deficiency in China [17], [18]. If we consider 25(OH)D levels below 15 ng/ml as deficiency, only 53.31% would have been non-deficient because most patients' 25(OH)D concentrations were between 10 and 15 ng/ml, with median PTH levels of 43.20±30.90. Several reports in China noted that vitamin D deficiency affects bone metabolism, especially bone formation, only when 25(OH)D levels became less than 10 ng/ml, instead of 20 ng/ml. Thus, we considered 16 ng/ml as a proper level of vitamin D for patients in the Shanghai area and recommended all age group patients, except the 50–59 years old group to increase their vitamin D uptake. We found that the variations of 25(OH)D2, 25(OH)D3 and total 25(OH)D serum concentrations did not strictly correspond to each other; the 25(OH)D3 level positively correlated with total 25(OH)D, but negatively correlated with 25(OH)D2. It has been reported that 25(OH)D3 will decrease after vitamin D2 supplementation likely due to competition for the 25-hydroxylase enzyme by vitamin D2[19]. 25-hydroxylase enzyme occupation may also be the explanation for the inconsistency among the correlations between vitamin D type serum concentrations and diseases. In general, age is considered a risk factor for vitamin D deficiency due to a decrease in vitamin D3 synthesis in the skin, and lower intakes of dietary vitamin D[20], [21], [22], [23]. But though a report from the US NHANES III (1988–1994) demonstrated an increasing prevalence of vitamin D deficiency and insufficiency with age [23], increasing contrary evidences are challenging this traditional view. Several studies have shown that the prevalence of vitamin D deficiency and insufficiency are higher instead of lower in younger than older people [24]. In the present study, our data also showed that vitamin D deficiency and insufficiency are most prevalent in the subject group over 90-year-olds, but such differences may be due to the selection of the participants in which patients less than 30 years old were excluded. In addition, other behavioral factors may also influence the serum vitamin D concentrations, such as indoor lifestyles, sunscreen overuse, less vitamin D supplementation and lower intakes of vitamin D, [25] while serum vitamin D levels also vary with seasons. 25(OH)D levels were lower in winter and summer than in spring and falls in our study. These results are partially different from other previous reports that vitamin D serum concentrations were lower in winter and spring [26], but in agreement with a previous analysis in China [27]. We suggest that in contrast to moderate climatic areas, the hot and humid summer in Shanghai must be taken into consideration, since most citizens and particularly elderly people, who are not in a healthy condition escape from the heat. Vitamin D remains relevant in high-TB burden settings and studies of TB patients in the tropics consistently show lower serum 25(OH)D in TB patients than in local healthy controls[28]. Vitamin D sufficiency has also been hypothesized to decrease TB infection risk after exposure, repress progression from latent to active TB and decrease the duration or improve the effectiveness of treatments as an adjunct to antimicrobial therapies [9], [29]. Our data showed a lower 25(OH)D3 and total 25(OH)D serum concentration in pulmonary disease patients, which further supports the potential role of vitamin D in infectious diseases. In our study, we also found that 25(OH)D levels of coronary heart disease and dyskinesia patients were significantly lower than the one of other patients. This is supported by Douglas et al., who reported that the incidence and mortality rates of coronary heart diseases showed a strong seasonal pattern with higher rates in winter, when vitamin D levels are lowest[30]. 25(OH)D serum levels of our hypertension patients were above the average, which is contrary to a report that increasing vitamin D uptake alleviates hypertension [31]. On the other hand, it is note-worthy that 25(OH)D2 serum levels of hypertension patient were lower than those of patients with other diseases and further investigations are necessary for an explanation at present.

### Conclusion

According to our study, we found that a large number of people are in need of vitamin D supplementation, no matter if we define insufficiency/deficiency as either 25(OH)D serum concentrations <20 ng/ml or <15 ng/ml. We also found that UVB exposure generates 10 times more 25(OH)D3 than diet or vitamin supplements can provide. Thus, we recommend that particularly over 65 years old patients in the Shanghai area should participate in more outdoor activities and obtain 600 IU vitamin D daily through sunlight exposure. By following our suggestion, patients could potentially improve their treatments of dyskinesia, coronary heart, lung and other diseases thereby improving their quality of life.
